# The Decline in Task Performance After Witnessing Rudeness Is Moderated by Emotional Empathy—A Pilot Study

**DOI:** 10.3389/fpsyg.2020.01584

**Published:** 2020-07-07

**Authors:** Gadi Gilam, Bar Horing, Ronny Sivan, Noam Weinman, Sean C. Mackey

**Affiliations:** ^1^Systems Neuroscience and Pain Laboratory, Division of Pain Medicine, Department of Anesthesiology, Perioperative, and Pain Medicine, School of Medicine, Stanford University, Palo Alto, CA, United States; ^2^School of Psychological Sciences, Tel Aviv University, Tel Aviv, Israel

**Keywords:** rudeness, empathy, task performance, social behavior, negative affect

## Abstract

Rude behaviors engulf societies across the world on a daily basis. Witnessing rudeness toward others increases negative affect and decreases performance in various tasks requiring behavioral and cognitive efforts, such as solving word puzzles or creative and flexible thinking. In this pilot study, we examined whether different levels of emotional empathy that may influence susceptibility to others’ distress, moderated the declined performance in several such tasks. The study was conducted online as a naturalistic setting for witnessing movie-clips portraying rudeness. We hypothesized that all participants will demonstrate decreased task performance following a rude compared to a neutral condition, but more so for those higher on emotional empathy. Results confirmed each of these hypotheses in one of two different cognitive tasks. Findings suggest that after witnessing rudeness, those higher on emotional empathy perform worse in cognitive tasks. While requiring replication in a larger sample size, empathic processing seems to be a potential moderator of the effect of rudeness on task performance.

## Introduction

Have you ever gone to the movies and had that person a couple of seats from you start talking loudly on the phone? It seems such rude behaviors have engulfed all levels of society, on a daily basis, and across the entire globe (Pearson and Porath, 2005; Truss, 2005; Schilpzand et al., 2016; Cortina et al., 2017). Rudeness is a behavioral expression of disrespect or lack of courtesy toward other people that breaches social norms of conduct. The societal implications of rudeness are considerable, since even minor acts of incivility may spiral to interpersonal conflict, increased aggression, and revenge (Bies and Tripp, 1996; Pearson and Porath, 2005; Forgas et al., 2011). In the workplace, rudeness can decrease productivity and performance in various tasks (Schilpzand et al., 2016) leading employers to incur substantial direct and indirect costs (Porath, 2015). In this context, regardless of whether a performance of interest is cognitive or behavioral, it refers to how well a person completes that certain task (Campbell, 1990).

On a personal level, victims of rudeness report experiencing distress and negative emotions, especially anger, fear, and sadness (Cortina et al., 2001; Porath and Pearson, 2012). In the aftermath of a rude event, victims also tend to engage in rumination (Porath et al., 2010)—recurrent thoughts about the event, its meanings, causes, and actual or alternate consequences, all of which may delay recovery from the event. A set of studies by Porath and Erez (2007, 2009) provides consistent evidence that even subtle operationalizations of rudeness lead to a decreased performance in various cognitive tasks, including word-puzzles, creativity, flexibility, and prosocial behaviors, such as helpfulness and sharing resources. Further findings indicate that rude and aggressive behaviors directly disrupt cognitive processes such as working-memory (Porath and Erez, 2007; Rafaeli et al., 2012) and induces negative affect (Porath and Erez, 2009)—a general aversive emotional state (Watson et al., 1988)—en route to diminished performance and prosociality.

As it seems, bystanders may also react emotionally and behaviorally to everyday incivility that occurs to other people in their surroundings. In many cases, emotions such as anger, contempt, and disgust may reflect the affective reactions to a perceived transgression inflicted by someone upon another person (Haidt, 2003). Indeed, anger was shown to be the most frequent emotional response to acts of incivility, and more commonly associated with sanctioned reactions (Phillips and Smith, 2004). However, being an observer, rather than being directly involved, may also evoke fear and, to a lesser extent, disgust. Remarkably Porath and Erez (2009) show that even witnessing rudeness toward another person, and not necessarily being the direct target of such behavior, led to increase in negative affect, which in fact mediated the relationship between witnessing rudeness and the decline in tasks assessing cognitive performance. They reasoned that witnessing rudeness might induce negative affect via several processes, which may subsequently disrupt cognitive processing and lead to the detrimental outcomes. People experience negative affect as a genuine response in the interest of others’ well-being. However, this might in fact reflect the possibility that people are selfishly focused on their own interests, and worry about the possibility that they might be next in line for such treatment (Fehr and Gächter, 2002). Another potential route contributing to increased negative affect is via the process of empathizing.

Empathy is an umbrella term referring to several related but distinct phenomena. Supported by findings from neuroscience, current accounts of empathy highlight three main components (Singer and Lamm, 2009; Shamay-Tsoory, 2011; Decety and Svetlova, 2012; Zaki and Ochsner, 2012): an *emotional* component, referring to taking on and sharing another person’s feelings; a *cognitive* component, referring to taking another’s perspective and representing their mental state; and a *motivational* component, referring to the concern one might have for another’s state and desiring to improve it (often referred to as compassion or sympathy). Emotional empathy is of particular relevance in the context of witnessing rude behaviors since the observers may vicariously share the emotional experience of the victim and experience personal distress themselves. Emotional empathy may therefore contribute to the increase in negative affect when witnessing rude behavior inflicted upon another person, and thus have a role in leading to poor task performance. Examining this moderating role of emotional empathy is therefore crucial to extend our understanding of how rude behaviors impact our society and which individuals might be at an increased risk for its negative implications. This is supported by the known findings that indicate the existence of individual differences in how people perceive and respond to rude behaviors (for review, see Cortina et al., 2017).

Here we aimed to examine whether emotional empathy will moderate the effect of witnessing rude behavior inflicted upon someone else on performance in several cognitive tasks. We assumed higher levels of emotional empathy lead to increased experience of negative affect when witnessing rudeness, and subsequently to more disruption in cognitive processing. Therefore, we hypothesized that people with higher levels of emotional empathy will exhibit poorer task performance after witnessing rudeness, compared to people lower on emotional empathy. Notably, rudeness has virus-like effects that can spread to uninvolved third parties (Foulk et al., 2016) and also contaminate one’s own perceptions and behaviors throughout the day (Woolum et al., 2017). Therefore, we aimed to examine whether the effects of witnessing rudeness can occur when watching an online video depicting rude behaviors toward others. This may have significant real-world implications in view of the *virality* of many such on line videos seen by millions worldwide (CBS News, 2015; Jerusalem Post, 2015; The Straits Times, 2015). The entire study was therefore conducted online. Participants first filled the emotional empathy scale (Mehrabian and Epstein, 1972). Participants were then counterbalanced between watching a short rude or neutral movie-clip, and subsequently completed two previously used cognitive performance tasks (Porath and Erez, 2007, 2009). We expected the rude movie-clip to have an overall negative effect on task performance, and that participants with higher empathy levels will demonstrate lower task performance levels compared to those with lower empathy levels.

## Materials and Methods

### Procedure

The study included two phases both of which required participants to log into the Qualtrics platform (http://www.qualtrics.com). In the first phase of the study, we published in social media and sent to various email lists an ad inviting people to participate in an experiment on memory and cognitive performance. People who logged into the Qualtrics platform first read an explanation that this was a first of a two-phase study, and then had to mark their informed consent to participate. They subsequently filled out basic demographic and contact information and then the emotional empathy scale (Mehrabian and Epstein, 1972). After 6 days of data collection, we calculated a median of the empathy score, and 3 days later sent each participant a personal email with a link to the second phase. Although it does not ensure that participants necessarily score at the low or high end of the scale (see below), the use of a median provided an empirical procedure (as previously used Rymarczyk et al., 2019) to *a priori* ensure that the distribution of empathy scores across our specific sample is similarly represented in both experimental conditions. We thus counterbalanced the conditions such that about half of the participants with an empathy score below the median and half of those with a score above the median received the rudeness experimental condition, while the other halves received the neutral control condition. In the second phase, we first instructed participants to watch a short movie-clip and then perform a memory test on the content of the clip. They subsequently performed two cognitive performance tasks. Following previous research (Porath and Erez, 2007, 2009), they were first administered an anagram task and then a brick task. We provided a thorough explanation of each task before participants actually began. We stopped data collection of the second phase after 26 days, and sent an email with the study debrief to all participants. In all phases, we conducted the study in accordance with the ethical principles of the Helsinki declaration for research on human subjects under the full responsibility of the authors. We report below how we determined our sample size, all data exclusions, all manipulations, and all measures in the study. Materials and data are available on the Open Science Framework^1^.

### Participants

The sample consisted of 112 individuals (59 females; ages 18–73, *M* = 28.37 ± 9.76, mean ± SD) who volunteered to participate. All participants had at least 12 years of education and a mother-tongue level proficiency for reading and writing Hebrew. A previous study on witnessing rudeness (Porath and Erez, 2009) examined an interaction effect of two factors based on a sample of *n* = 80. The number of participants was therefore *a priori* aimed for 120 participants. We needed to excluded 38 participants based on three criteria: 35 did not complete the second phase, two performed by mistake both conditions of the second phase, and one participant did not pass the manipulation-test question.

### Emotional Empathy

The emotional empathy questionnaire was developed and validated by Mehrabian and Epstein (1972) reflecting the tendency to respond emotionally to another person’s emotional experience. The questionnaire includes 33 items, 17 of which are negatively phrased, representing various aspects of emotional empathy such as susceptibility to emotional contagion (e.g., “The people around me have a great influence on my moods”), emotional responsiveness to others’ positive and negative emotional experiences (e.g., “I like to watch people open presents”), and sympathetic tendencies (e.g., “Little children sometimes cry for no apparent reason”). Participants are asked to rate each item on a −4 (very strong disagreement) to 0 (neutral) to +4 (very strong agreement) scale. The total empathy score sums all items after reversing the scores for the negative items, resulting in a potential range of −132 (complete disagreement) to +132 (complete agreement). Here we administered a previously used Hebrew translation of the questionnaire (Bar, 1999). For the initial *N* = 150 sample: Cronbach’s 0.82 = α, median = 29.00, *M* = 30.19 ± 22.42. For the final *N* = 112 sample: Cronbach’s 0.79 = α, median = 28.00, *M* = 30.13 ± 23.93. The range for both was the same, with a minimum of −15, and a maximum of 92. These values are in line with those previously reported in the literature (Milders et al., 2003; Hampson et al., 2008; Gao et al., 2016). The median split of the final sample generated a sample specific lower empathy group (*M* = 10.65 ± 10.80, 95% CI = [7.85, 13.45]), which was significantly different than a higher empathy group (*M* = 50.69 ± 15.04, 95% CI = [46.72, 54.67]; *t*_df = 110_ = 16.23, *p* < 0.001, *Cohen’s d* = 3.06). To note, the average response score for the lower group reflects a tendency for a neutral response to the questionnaire items (*M* = 0.32 ± 0.33, 95% CI = [0.23, 0.40]), while for the higher group, it reflects a slight agreement (*M* = 1.54 ± 0.46, 95% CI = [1.42, 1.66]). Nevertheless, these artificially generated groups were used only for allocation of the manipulation conditions, and the actual analyses used the raw empathy sum score.

### Manipulation

To portray a situation of rudeness, we used a movie-clip of a real event captured by a hand-held phone. The movie (1:48 min long) portrayed a situation in a medical services waiting room during which a person is cursing a nurse in front of by-standers. As control, we used a neutral movie-clip (1:08 min long) from a TV-show portraying a polite social-interaction between new neighbors introducing themselves to each other. We conducted a pre-test to validate that the two movie-clips reflected a rude and non-rude interaction, respectively. Eight participants from a similar sampling frame as the study sample saw each clip in a counterbalanced fashion, and subsequently rated the level of rudeness portrayed within the clip on a 0 = no rudeness to 7 = very rude scale. All participants rated the polite clip as 0 and the rude clip as 7.

Since participants saw the movie-clip in an uncontrolled home environment, we added a multiple-choice memory test to ensure their engagement, with questions such as “where did the scene occur?” The first of the six memory questions for the neutral clip and of the seven memory questions for the rudeness clip was in fact a manipulation test, asking whether the clip portrayed a rude, jealous, or polite social-interaction, and served as exclusion criteria. For the other memory questions, no participant had more than two wrong answers, and there was no difference in average percent of wrong answers between the two clips (rude = 9.77 ± 10.83%, 95% CI = [6.92, 12.62]; neutral = 7.04 ± 9.64%, 95% CI = [4.41, 9.67]; t_df = 110_ = 1.41, *p* = 0.162, *Cohen’s d* = 0.27).

### Task Performance

We measured task performance based on the same two measures used by previous studies on the effects of rudeness on performance (Porath and Erez, 2007, 2009). We used a moderately difficult anagram task consisting of 10 anagrams as the first task and the number of anagrams correctly solved in 5 min was one measure of task performance. We conducted a pre-test to examine and choose the set of anagrams. Nine participants from a similar sampling frame as the study sample solved 30 anagrams of various difficulties in 15 min. We followed Organ (1977) model to choose the final 10 anagrams (Table 1), which comprised of two easy anagrams (>85% correct answer), five moderate anagrams (40–60% correct), and three hard anagrams (<35% correct).

**TABLE 1:** The 10 anagrams used for the anagram performance task.

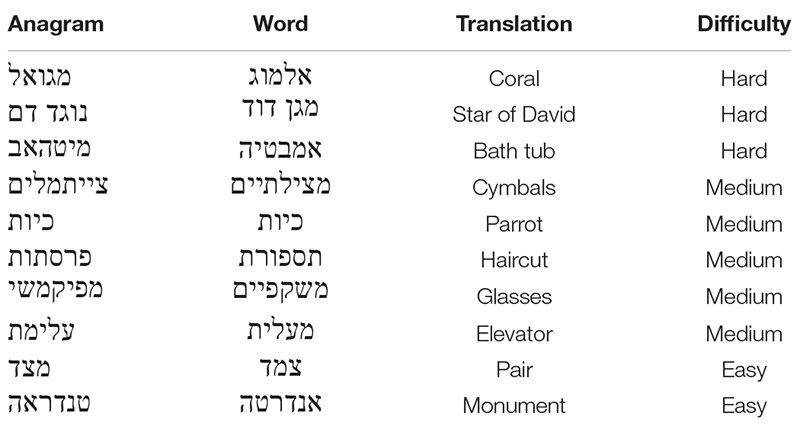

The second task was writing down as many uses of a brick one could think of in one minute. The number of brick-uses produced during the task, termed fluency, was another measure of task performance. Three of the authors blind to the manipulation condition independently validated that there were no repetitions in brick uses within subjects (e.g., building and constructing) and if there was disagreement, the forth author made a final decision based on the majority. Participants suggested 525 brick uses, and we discarded 19 of them as invalid repetitions.

Measures of creativity and flexibility were also based on performance in the brick-uses task, and derived following the procedures implemented by previous studies (Porath and Erez, 2007, 2009). Three of the authors blind to the manipulation condition independently rated the creativity and flexibility of the brick uses on 1–7 scale where 1 = low, 4 = medium, and 7 = high. The low and high ends of the scale were anchored using the same examples as in Porath and Erez (2007, 2009). Examples for high creativity ratings were “hang in museum and call it abstract art” and “sell on eBay,” while examples for low creativity ratings were “build a house” and “door stop.” Examples for high flexibility ratings were those cases were brick uses included a variety of categories such as building, weights, interior decoration, weapon, and etcetera. For low flexibility, categories included, for example, only building, though building two types of things such as walls or fences compared to houses or buildings was a little more flexible. We assessed reliability of ratings using intraclass correlation coefficients (ICCs) which indicated that averaging ratings across raters was appropriate: ICC(2,*k* = 3) was 0.74 and 0.80 for creativity and flexibility, respectively.

## Results

Table 2 presents the descriptive results of gender distribution, emotional empathy scores, and scores in each of the four dependent variables, per the two manipulation conditions. Correlation between the four dependent variables is presented in Table 3. While gender distribution was equal across manipulation conditions, a *post hoc t*-test confirmed the apparent gender difference in emotional empathy (Mestre et al., 2009; Christov-Moore et al., 2014) indicating females (*M* = 40.97 ± 22.84, 95% CI = [35.02, 46.92]) had higher emotional empathy levels then males (*M* = 18.45 ± 19.23, 95% CI = [13.15, 23.75]; *t*_110_ = 5.61, *p* < 0.001, *Cohen’s d* = 1.07). To control for the potential bias related to the gender distribution across conditions, we performed regression analyses with the Emotional Empathy (using the raw score centered to the mean), Manipulation (rude/polite movie clip), as well as Gender (male, female) as between-subject factors. In the first step of the regression (Model 1), we entered these three factors to examine their main effects. In the second step (Model 2), we entered three regressors that were computed as the product of each two factors, in order to examine the three interaction effects. In the third step (Model 3), we entered a regressor that was computed as the product of all three factors to examine the three-way interaction. We conducted this analysis for each of the dependent variables, namely in the anagram task (Supplementary Table S1), and in the brick task (Supplementary Table S3 for fluency, Supplementary Table S5 for creativity, and Supplementary Table S7 for flexibility). The same regression analyses were also repeated without the Gender factor (Supplementary Tables S2, S4, S6, S8).

**TABLE 2:** Descriptive summary of results per the two manipulation conditions.

		Female	Emotional empathy	Anagram score	Fluency score	Creativity score	Flexibility score
	*N*	(#, %)	(mean ± stdv, min:max)	(mean ± stdv, min:max)	(mean ± stdv, min:max)	(mean ± stdv, min:max)	(mean ± stdv, min:max)
Rude	58	30, 51.72	30.19 ± 23.08, −15:78	5.05 ± 1.91, 1:9	4.35 ± 1.88, 0:9	2.98 ± 1.14, 0:5.67	3.13 ± 1.16, 0:6
Polite	54	29, 53.70	30.44 ± 25.05, −11:92	6.15 ± 2.09, 2:10	4.70 ± 2.09, 2:8	3.19 ± 1.27, 1:6	3.27 ± 1.16, 1:5.67
Total	112	59, 52.68	30.31 ± 23.94, −15:92	5.58 ± 2.06, 1:10	4.52 ± 1.70, 0:9	3.08 ± 1.20, 0:6	3.19 ± 1.16, 0:6

**TABLE 3:** Correlation between dependant measures.

	Anagram score	Fluency score	Creativity score	Flexibility score
Anagram score				
Fluency score	0.040			
Creativity score	0.072	0.730***		
Flexibility score	0.135	0.735***	0.920***	

Fluency, creativity, and flexibility scores were derived from the same brick-uses task. ***p < 0.001.

**FIGURE 1:**
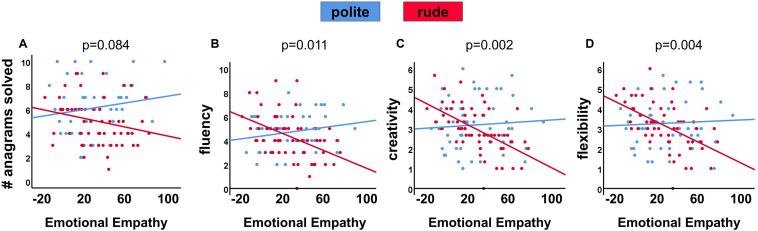
Graphs depicting the interactions between emotional empathy and the rudeness manipulation. **(A)** The number of solved anagrams (Beta = −0.254, *p* = 0.084). **(B)** Fluency scores (Beta = −0.368, *p* = 0.011). **(C)** Creativity scores (Beta = −0.466, *p* = 0.002). **(D)** Flexibility scores (Beta = −0.419, *p* = 0.004). Higher emotional empathy in the rude condition (red) was associated with worse performance (*R*^2^_#anagram_ = 0.057, *R*^2^_fluency_ = 0.201, *R*^2^_creativity_ = 0.324, *R*^2^_flexibility_ = 0.281), while no association was observed in the polite condition (blue: *R*^2^_#anagram_ = 0.032, *R*^2^_fluency_ = 0.042, *R*^2^_creativity_ = 0.005, *R*^2^_flexibility_ = 0.003).

We first examined performance in the anagram task (Supplementary Table S1). In line with our hypothesis, there was a main effect of movie-clip manipulation (Beta = −0.268, *t* = −2.888, *p* = 0.005), indicating better performance following the polite (*M* = 6.15 ± 2.09, 95% CI = [5.59, 6.71], *N* = 54), compared to the rude movie-clip (*M* = 5.05 ± 1.90, 95% CI = [4.56, 5.54], *N* = 58). Unlike our hypothesis, the interaction effect of the movie-clip manipulation with emotional empathy was not significant (Beta = −0.254, *t* = −1.746, *p* = 0.084; Figure 1A). This effect was significant when gender was not included in the analysis (*p* = 0.029; Supplementary Table S2).

We next examined each of the three measures derived from the brick-uses task, namely, fluency (Supplementary Table S3), creativity (Supplementary Table S5), and flexibility (Supplementary Table S7). Unlike our hypothesis, there was no main effect for the movie-clip manipulation in any of these dependent variables (fluency: Beta = −0.109, *t* = −1.152, *p* = 0.252; creativity: Beta = −0.090, *t* = −0.971, *p* = 0.333; flexibility: Beta = −0.064, *t* = −0.692, *p* = 0.491). However, in line with our hypothesis, in all three cases, the interaction effect of the movie-clip manipulation with emotional empathy was significant (fluency: Beta = −0.368, *t* = −2.584, *p* = 0.011; creativity: Beta = −0.466, *t* = −3.256, *p* = 0.002; flexibility: Beta = −0.419, *t* = −2.928, *p* = 0.004), and remained so in the final model that included also the three-way interaction of manipulation, emotional empathy and gender (*p*_fluency_ = 0.031, *p*_creativity_ = 0.007, *p*_flexibility_ = 0.007). In line with our hypothesis, all these interactions indicated the same pattern of result (Figures 1B–D): higher emotional empathy in the rude condition was associated with worse performance (*R*^2^_fluency_ = 0.201, *R*^2^_creativity_ = 0.324, *R*^2^_flexibility_ = 0.281), while no association was observed in the polite condition (*R*^2^_fluency_ = 0.042, *R*^2^_creativity_ = 0.005, *R*^2^_flexibility_ = 0.003). These effects were significant also when gender was not included in the analysis (*p*_fluency_ < 0.001, *p*_creativity_ = 0.001, *p*_flexibility_ = 0.001; Supplementary Tables S4, S6, S8). Given that these three measures are derived from the same task and are strongly correlated (Table 3), it is not surprising that they produce very similar results.

As an additional and final step, since adding the Gender factor introduced 11 more comparison per each of the four dependent variables that were not originally planned, we re-examined the above results by applying a false discovery rate (FDR) to control for multiple comparisons (i.e., 16 comparisons per dependent variable). The movie-clip manipulation by emotional empathy interaction effect for the creativity score survived the correction threshold at q(FDR) < 0.05 (*p*_critical_ = 0.002). Two of the above reported effects, a similar interaction effect for flexibility and the main effect for number of anagrams solved, passed a slightly more lenient threshold of q(FDR) < 0.08 (*p*_critical_ = 0.007 and *p*_critical_ = 0.005, respectively). The fluency interaction effect did not survive the correction threshold. All significant effects in models without the gender factor (i.e., five comparisons per dependent variable) survived the correction threshold at q(FDR) < 0.05 (*p*_critical_ = 0.029, *p*_critical_ = 0.000, *p*_critical_ = 0.015, and *p*_critical_ = 0.012, respectively).

## Discussion

This study examined whether the influence of witnessing rudeness on task performance depends on different levels of emotional empathy, which may impact the susceptibility to others’ distress. Previous research consistently showed that being a target of or even just witnessing rude behaviors, leads to numerous negative outcomes, including an increase in negative affect, disruption of cognitive processing, and decreased cognitive performance (Schilpzand et al., 2016). Our study requires replication in a larger sample size, and with better control for factors such as gender, which was not planned to be included and led to increased number of statistical comparisons. Although only one metric of cognitive performance survived correction for multiple comparisons, findings generally indicated an overall convergence with the direction of our hypothesis across all measures, suggesting that after watching a movie-clip of a rude event, participants with higher levels of emotional empathy performed worse compared to participants with lower levels of emotional empathy. In the anagram task, we found a general negative impact of rudeness on performance above and beyond empathy levels, though at a slitley more lenient statistical threshold. However, differences between the two movie-clips used in each condition (see below) might have confounded this specific finding and should be addressed in future replications. The preliminary findings extended previous research by indicating that witnessing rudeness may have its negative impact on task performance, depending on a person’s level of emotional empathy.

Within a negative context such as witnessing rudeness, emotional empathy can lead people to experience the distress of the victim. Since previous studies demonstrate that negative affect mediated the realtionship between witnessing rudeness and task performance (Porath and Erez, 2009; Schilpzand et al., 2016), we assumed individuals with higher levels of emotional empathy will experience more personal distress, and thus as demonstrated, perform worse in the cognitive tasks. However, limitations apply since participants’ negative affect was not measured. Future studies need to address this gap by recording participant’s self reports and/or physiological respsonse, such as heart-rate or galvanic skin response. This may support the link between emotional empathy, negative affect, and cognitive processing.

Other empathic components such as cognitive empathy, also known as perspective taking, may play a different role in impacting people’s cognitive processing following a rude event. At least from the perspective of the victim, being able to take the perspective of the perpetrator was shown to attenuate the effect of rude and aggressive behaviors on task performance (Rafaeli et al., 2012). Using a questionnaire that measures multiple components of empathy, such as the Interpersonal Reactivity Index (Davis, 1980), might reveal the inter-relationships between these different components, and their role in mediating or moderating the effect of rudeness on task performance.

Empathy broadly defined is generally regarded as an adaptive socio-emotional capability that is crucial for maintaining relationships and for motivating prosocial behaviors (Decety and Svetlova, 2012). This has linked empathy with morality, and strengthened the notion that empathy should be cultivated no matter what—the more the better. However, in the specific context of the current study, our findings indicate that higher levels of emotional empathy may have detrimental effects on cognitive performance. On the other hand, if being highly empathic to the wrong doings inflicted toward some other person may spark anger and motivate one to reach out and assist that person, the toll on cognitive processing might be worthwhile. This has not been tested here and should be addressed in future studies. Ultimately, the relationship between empathy and morality is complex and equivocal, and the current findings contribute to the ongoing debate (Decety and Cowell, 2014; Bloom, 2017a, b; Zaki, 2017).

Another important contribution of our study relates to the ecological settings in which we carried out our study. Granted, previous studies used scripted movie-clips to induce the negative impact of rudeness (Foulk et al., 2016; Woolum et al., 2017). These studies were able to show that rudeness may spread like a virus, contaminating one’s own perceptions and behaviors following the event, as well as spreading to uninvolved third parties. Here we took another step forward by examining whether the effects of witnessing rudeness can also be mediated by watching at home an online movie-clip depicting a real unedited rude behavior that was captured using a standard phone. This is of particular importance since many such movie-clips become *viral* and are seen by millions of people throughout the world (CBS News, 2015; Jerusalem Post, 2015; The Straits Times, 2015). Notably, this was generally found for the anagram task, while for the brick task, the effects were observed for the people with higher emotional empathy scores. This suggests, as might be expected, that witnessing rudeness in an online video might have less of an impact compared to actually being on the scene. Indeed, video-mediated communication may lack the richness of physical, visual, and auditory cues that are present during interpersonal interactions (Daft and Lengel, 1986; Kock, 2005). However, it is still important to warn people that watching such movie-clips online may incur some emotional and cognitive costs, and emphasize that if they become viral, they may further perpetuate the negative effects of rudeness.

The primary limitation of our findings is that they require replication with a larger sample size. Thus said, we do replicate in those with higher empathy scores the general findings of numerous studies showing the negative impact of rudeness on task performance (Schilpzand et al., 2016) and thus it is reasonable to assume our findings are not coincidental. Notably, we *a priori* aimed for and indeed obtained a sample size larger than that used in a previous study (Porath and Erez, 2009). However, the attrition rate was higher than we anticipated. This is of particular relevance to the main effect of manipulation in the anagram task. Notably, the effect of gender, which we did not initially plan to include in the analysis, reduced the strength of our findings (see Supplementary Tables S1–S4). Also to note that we gave participants only 5 min for the anagram task as compared to 10 min in previous studies (Porath and Erez, 2007, 2009) and if participants would have had the extra time, it might have revealed stronger effects.

Another important limitation refers to the characteristics of the two movie-clips used for the manipulation, which differed in length, quality, editing style (e.g., shot angle, speed of scene changes), environmental surrounding, as well as to the fact that the perpetrator of rudeness is a man targeting a woman. This can explain the fact that most main effects of manipulation resulted in null findings, and should be controlled and/or examined in future studies. Nevertheless, the key finding of this pilot study is independent from differences between movies since all participants within the rudeness condition saw the exact same movie. Notably, the rudeness effect did not have an impact on those with lower empathy in the brick task, and even suggesting a potential reverse effect. While we should be cautious in speculating, future studies may aim to examine this issue directly, for example, by also measuring boredom as an additional potential moderator of the effects. Boredom could be relevant since the neutral movie-clip might have generated a detached responsivity to the study, and decreased engagement in the performance tasks. Similarly to consider, in real-life one may decide to interrupt the video and avoid the exposure to neutral or rude movie clips, thus limiting the naturalistic settings of this in-home study.

Finally, limited generalizability is also a factor to consider in regard of: (1) types of performances, for example, to include also working memory or math capabilities (Porath and Erez, 2007; Giumetti et al., 2013), (2) types of rude behaviors, for example, whether expressed through language, bodily decorum, or actions (Smith et al., 2010), and (3) type of interpersonal situations, for example, witnessed through movie-clips at home, or in the actual presence of the event. Extending future studies to the variability within these factors may help understand the boundaries of the effects of rudeness on performance in general, and of the moderating effect of emotional empathy in particular.

Taken together, while requiring replication, these preliminary findings suggest that the decline in task performance after witnessing rudeness is differentially moderated by levels of emotional empathy, with higher emotional empathy associated with worse performance. Findings also illustrate that simply watching a short movie-clip online at home, or potentially anywhere else, may lead to this detrimental effect, particularly if one has higher levels of emotional empathy. While socio-cultural norms dictate what might be considered as incivility in different places around the world, curbing rudeness and cultivating appropriate interpersonal relationships is a standard everywhere. It might also be important to reconsider whether to watch that viral movie portraying rudeness popping up in our social media feed, especially if one is prone to an emotionally empathic response.

## Data Availability Statement

Materials and data are available at https://osf.io/fh6pb/.

## Ethics Statement

Ethical review and approval was not required for the study on human participants in accordance with the local legislation and institutional requirements. The patients/participants provided their written informed consent to participate in this study.

## Author Contributions

GG, BH, RS, and NW designed the study. BH, RS, and NW collected the data. GG analyzed and interpreted the data, and wrote the manuscript. All authors contributed to analyzing, interpreting, and reviewing the manuscript.

## Conflict of Interest

The authors declare that the research was conducted in the absence of any commercial or financial relationships that could be construed as a potential conflict of interest.

## Footnotes

1 https://osf.io/fh6pb/
