# Supplementary material for: The Decline in Task Performance After Witnessing Rudeness Is Moderated by Emotional Empathy—A Pilot Study
Source: Front Psychol. 2020 Jul 7;11:1584. doi: 10.3389/fpsyg.2020.01584 (PMC7358519; doi:10.3389/fpsyg.2020.01584)
Supplement: Supplementary file 1 [file Table_1.DOCX]

**Title:**

The decline in task performance after witnessing rudeness is moderated by emotional empathy – a pilot study

**Authors:**

Gadi Gilam^1,2^, Bar Horing^2^, Ronny Sivan^2^, Noam Weinman^2^, Sean C. Mackey^1^

**Affiliation:**

^1^ Stanford University School of Medicine, Department of Anesthesiology, Perioperative, and Pain Medicine, Division of Pain Medicine, 1070 Arastradero Road, Suite 200, MC 5596, Palo Alto, CA 94304, USA.

^2^ Tel Aviv University, School of Psychological Sciences, P.O. Box 39040, Tel Aviv 69978, Israel

**Corresponding author:**

Gadi Gilam, Ph.D.

Systems Neuroscience and Pain Laboratory, Division of Pain Medicine, Department of Anesthesiology, Perioperative and Pain Medicine, Stanford University School of Medicine, 1070 Arastradero Road, Suite 200, MC 5596, Palo Alto, CA, 94304, USA

Phone: (650) 353-8331. Email: [gadi.gilam@gmail.com](mailto:gadi.gilam@gmail.com) / gadi.gilam@stanford.edu

Materials and data are available on the Open Science Framework (<https://osf.io/fh6pb/>).

**Supplemental Results**

**Table S1:** Regression analysis with the anagram task score as the dependent variable

| Model | | Unstandardized Coefficients | | Standardized Coefficients | t | Sig. | R^2^ | Adj. R^2^ | Collinearity Statistics | | 95% CI for B | | Correlations | | |
| --- | --- | --- | --- | --- | --- | --- | --- | --- | --- | --- | --- | --- | --- | --- | --- |
|  |  | B | Std. Error | Beta |  |  |  |  | Tolerance | VIF | LB | UP | Zero-order | Partial | Part |
| 1 | (Constant) | 6.218 | .358 |  | 17.348 | .000 | .072 | .047 |  |  | 5.508 | 6.929 |  |  |  |
|  | Empathy (raw score) | .000 | .009 | .004 | .035 | .972 |  |  | .778 | 1.286 | -.018 | .018 | -.017 | -.003 | -.003 |
|  | Manipulation (rude/polite) | -1.099 | .381 | -.268 | -2.888 | .005 |  |  | 1.000 | 1.000 | -1.853 | -.345 | -.267 | -.268 | -.268 |
|  | Gender (male/female) | -.131 | .432 | -.032 | -.303 | .763 |  |  | .777 | 1.287 | -.987 | .725 | -.028 | -.029 | -.028 |
| 2 | (Constant) | 5.998 | .475 |  | 12.619 | .000 | .144 | .095 |  |  | 5.055 | 6.940 |  |  |  |
|  | Empathy | -.006 | .019 | -.066 | -.306 | .760 |  |  | .175 | 5.702 | -.042 | .031 | -.017 | -.030 | -.028 |
|  | Manipulation | -1.104 | .591 | -.269 | -1.867 | .065 |  |  | .393 | 2.544 | -2.276 | .069 | -.267 | -.179 | -.169 |
|  | Gender | -.160 | .652 | -.039 | -.245 | .807 |  |  | .324 | 3.083 | -1.452 | 1.132 | -.028 | -.024 | -.022 |
|  | Empathy^Manipulation | -.032 | .018 | -.254 | -1.746 | .084 |  |  | .384 | 2.602 | -.068 | .004 | -.157 | -.168 | -.158 |
|  | Gender^Manipulation | .156 | .858 | .034 | .181 | .856 |  |  | .238 | 4.207 | -1.546 | 1.857 | -.210 | .018 | .016 |
|  | Gender^Empathy | .035 | .019 | .292 | 1.862 | .065 |  |  | .332 | 3.013 | -.002 | .072 | .125 | .179 | .168 |
| 3 | (Constant) | 5.919 | .529 |  | 11.198 | .000 | .145 | .087 |  |  | 4.871 | 6.967 |  |  |  |
|  | Empathy | -.011 | .024 | -.129 | -.457 | .649 |  |  | .103 | 9.741 | -.059 | .037 | -.017 | -.045 | -.041 |
|  | Manipulation | -.997 | .668 | -.243 | -1.493 | .138 |  |  | .311 | 3.219 | -2.322 | .327 | -.267 | -.145 | -.135 |
|  | Gender | -.108 | .671 | -.026 | -.161 | .873 |  |  | .308 | 3.244 | -1.439 | 1.223 | -.028 | -.016 | -.015 |
|  | Empathy^Manipulation | -.023 | .030 | -.187 | -.776 | .440 |  |  | .141 | 7.102 | -.083 | .036 | -.157 | -.076 | -.070 |
|  | Gender^Manipulation | .100 | .877 | .022 | .114 | .910 |  |  | .230 | 4.354 | -1.639 | 1.838 | -.210 | .011 | .010 |
|  | Gender^Empathy | .042 | .029 | .356 | 1.471 | .144 |  |  | .141 | 7.105 | -.015 | .099 | .125 | .143 | .133 |
|  | Empathy^Manipulation^Gender | -.013 | .038 | -.074 | -.348 | .729 |  |  | .182 | 5.481 | -.088 | .062 | -.097 | -0.34 | -.032 |

**Table S2:** Regression analysis with the anagram task score as the dependent variable, but without the Gender factor

| Model | | Unstandardized Coefficients | | Standardized Coefficients | t | Sig. | R^2^ | Adj. R^2^ | Collinearity Statistics | | 95% CI for B | | Correlations | | |
| --- | --- | --- | --- | --- | --- | --- | --- | --- | --- | --- | --- | --- | --- | --- | --- |
|  |  | B | Std. Error | Beta |  |  |  |  | Tolerance | VIF | LB | UP | Zero-order | Partial | Part |
| 1 | (Constant) | 6.148 | .273 |  | 22.552 | .000 | .072 | .055 |  |  | 5.608 | 6.689 |  |  |  |
|  | Empathy (raw score) | -.002 | .008 | -.019 | -.202 | .840 |  |  | 1.000 | 1.000 | -.017 | .014 | -.017 | -.019 | -.0191 |
|  | Manipulation (rude/polite) | -1.097 | .379 | -.267 | -2.895 | .005 |  |  | 1.000 | 1.000 | -1.848 | -.346 | -.267 | -.267 | -.267 |
| 2 | (Constant) | 6.146 | .268 |  | 22.945 | .000 | .112 | .087 |  |  | 5.615 | 6.677 |  |  |  |
|  | Empathy | .015 | .011 | .173 | 1.383 | .170 |  |  | .523 | 1.913 | -.006 | .036 | -.017 | .132 | .125 |
|  | Manipulation | -1.097 | .372 | -.267 | -2.947 | .004 |  |  | 1.000 | 1.000 | -1.835 | -.359 | -.267 | -.273 | -.267 |
|  | Empathy^Manipulation | -.035 | .016 | -.278 | -2.217 | .029 |  |  | .523 | 1.913 | -.066 | -.004 | -.157 | -.209 | -.201 |

**Table S3:** Regression analysis with fluency as the dependent variable

| Model | | Unstandardized Coefficients | | Standardized Coefficients | t | Sig. | R^2^ | Adj. R^2^ | Collinearity Statistics | | 95% CI for B | | Correlations | | |
| --- | --- | --- | --- | --- | --- | --- | --- | --- | --- | --- | --- | --- | --- | --- | --- |
|  |  | B | Std. Error | Beta |  |  |  |  | Tolerance | VIF | LB | UP | Zero-order | Partial | Part |
| 1 | (Constant) | 4.873 | .300 |  | 16.236 | .000 | .042 | .016 |  |  | 4.278 | 5.467 |  |  |  |
|  | Empathy (raw score) | .008 | .008 | .113 | 1.057 | .293 |  |  | .778 | 1.286 | -.023 | .007 | -.156 | -.101 | -.100 |
|  | Manipulation (rude/polite) | -.367 | .319 | -.109 | -1.152 | .252 |  |  | 1.000 | 1.000 | -.999 | .264 | -.106 | -.110 | -.109 |
|  | Gender (male/female) | -.312 | .362 | -.092 | -.864 | .389 |  |  | .777 | 1.287 | -1.029 | .404 | -.143 | -.083 | -.081 |
| 2 | (Constant) | 4.501 | .382 |  | 11.772 | .000 | .184 | .137 |  |  | 3.743 | 5.260 |  |  |  |
|  | Empathy | .001 | .001 | .021 | .099 | .921 |  |  | .175 | 5.702 | -.028 | .031 | -.156 | .010 | .009 |
|  | Manipulation | .189 | .476 | .056 | .397 | .692 |  |  | .393 | 2.544 | -.754 | 1.132 | -.106 | .039 | .035 |
|  | Gender | .212 | .524 | .063 | .405 | .686 |  |  | .324 | 3.083 | -.827 | 1.252 | -.143 | .040 | .036 |
|  | Empathy^Manipulation | -.038 | .015 | -.368 | -2.584 | .011 |  |  | .384 | 2.602 | -.067 | -.009 | -.355 | .245 | -.228 |
|  | Gender^Manipulation | -1.000 | .690 | -.262 | -1.449 | .150 |  |  | .238 | 4.207 | -2.369 | .368 | -.293 | .140 | -.128 |
|  | Gender^Empathy | .013 | .015 | .132 | .860 | .392 |  |  | .332 | 3.013 | -.017 | .043 | -.043 | .084 | .076 |
| 3 | (Constant) | 4.646 | .424 |  | 10.953 | .000 | .188 | .134 |  |  | 3.805 | 5.487 |  |  |  |
|  | Empathy | .011 | .020 | .162 | .587 | .559 |  |  | .103 | 9.741 | -.027 | .050 | -.156 | -.057 | .052 |
|  | Manipulation | -.006 | .536 | -.002 | -.011 | .991 |  |  | .311 | 3.219 | -1.069 | 1.057 | -.106 | -.001 | -.001 |
|  | Gender | .117 | .539 | .035 | .218 | .828 |  |  | .308 | 3.244 | -.951 | 1.185 | -.143 | .021 | .019 |
|  | Empathy^Manipulation | -.053 | .024 | -.516 | -2.193 | .031 |  |  | .141 | 7.102 | -.101 | -.005 | -.355 | -.210 | -.194 |
|  | Gender^Manipulation | -.898 | .704 | -.235 | -1.276 | .205 |  |  | .230 | 4.354 | -2.293 | .497 | -.293 | -.124 | -.113 |
|  | Gender^Empathy | -.001 | .023 | -.010 | -.043 | .966 |  |  | .141 | 7.105 | -.047 | .045 | -.043 | -.004 | -.004 |
|  | Empathy^Manipulation^Gender | .024 | .030 | .164 | .794 | .429 |  |  | .182 | 5.481 | -.036 | .084 | -.209 | .078 | .070 |

**Table S4:** Regression analysis with fluency as the dependent variable, but without the Gender factor

| Model | | Unstandardized Coefficients | | Standardized Coefficients | t | Sig. | R^2^ | Adj. R^2^ | Collinearity Statistics | | 95% CI for B | | Correlations | | |
| --- | --- | --- | --- | --- | --- | --- | --- | --- | --- | --- | --- | --- | --- | --- | --- |
|  |  | B | Std. Error | Beta |  |  |  |  | Tolerance | VIF | LB | UP | Zero-order | Partial | Part |
| 1 | (Constant) | 4.705 | .229 |  | 20.551 | .000 | .036 | .018 |  |  | 4.251 | 5.159 |  |  |  |
|  | Empathy (raw score) | -.011 | .007 | -.156 | -1.663 | .099 |  |  | 1.000 | 1.000 | -.024 | .002 | -.156 | -.157 | -.156 |
|  | Manipulation (rude/polite) | -.362 | .318 | -.107 | -1.137 | .258 |  |  | 1.000 | 1.000 | -.992 | 2.69 | -.106 | -.108 | -.107 |
| 2 | (Constant) | 4.702 | .216 |  | 21.814 | .000 | .153 | .130 |  |  | 4.275 | 5.129 |  |  |  |
|  | Empathy | .012 | .009 | .171 | 1.395 | .166 |  |  | .523 | 1.913 | -.005 | .029 | -.156 | .133 | .124 |
|  | Manipulation | -.362 | .300 | -.107 | -1.208 | .230 |  |  | 1.000 | 1.000 | -.956 | .232 | -.106 | -.115 | -.107 |
|  | Empathy^Manipulation | -.049 | .013 | -.474 | -3.869 | .000 |  |  | .523 | 1.913 | -.074 | -.024 | -.355 | -.349 | -.343 |

**Table S5:** Regression analysis with creativity in the brick task as the dependent variable

| Model | | Unstandardized Coefficients | | Standardized Coefficients | t | Sig. | R^2^ | Adj. R^2^ | Collinearity Statistics | | 95% CI for B | | Correlations | | |
| --- | --- | --- | --- | --- | --- | --- | --- | --- | --- | --- | --- | --- | --- | --- | --- |
|  |  | B | Std. Error | Beta |  |  |  |  | Tolerance | VIF | LB | UP | Zero-order | Partial | Part |
| 1 | (Constant) | 3.330 | .209 |  | 15.920 | .000 | .070 | .044 |  |  | 2.915 | 3.744 |  |  |  |
|  | Empathy (raw score) | .009 | .005 | .176 | 1.677 | .096 |  |  | .778 | 1.286 | -.019 | .002 | -.229 | -.159 | -.156 |
|  | Manipulation (rude/polite) | -.216 | .222 | -.090 | -.971 | .333 |  |  | 1.000 | 1.000 | -.656 | .224 | -.087 | -.093 | -.090 |
|  | Gender (male/female) | -.267 | .252 | -.112 | -1.060 | .292 |  |  | .777 | 1.287 | -.767 | .232 | -.193 | -.101 | -.098 |
| 2 | (Constant) | 3.409 | .272 |  | 12.532 | .000 | .174 | .126 |  |  | 2.870 | 3.949 |  |  |  |
|  | Empathy | .009 | .011 | .178 | .842 | .402 |  |  | .175 | 5.702 | -.012 | .030 | -.229 | .082 | .075 |
|  | Manipulation | -.289 | .338 | -.121 | -.855 | .394 |  |  | .393 | 2.544 | -.961 | .382 | -.087 | -.083 | -.076 |
|  | Gender | -.401 | .373 | -.167 | -1.074 | .285 |  |  | .324 | 3.083 | -1.140 | .339 | -.193 | -.104 | -.095 |
|  | Empathy^Manipulation | -.034 | .010 | -.466 | -3.256 | .002 |  |  | .384 | 2.602 | -.054 | -.013 | -.385 | -.303 | -.289 |
|  | Gender^Manipulation | .131 | .491 | .049 | .267 | .790 |  |  | .238 | 4.207 | -.843 | 1.105 | -.230 | .026 | .024 |
|  | Gender^Empathy | -.002 | .011 | -.022 | -.142 | .888 |  |  | .332 | 3.013 | -.023 | .020 | -.144 | -.014 | -.013 |
| 3 | (Constant) | 3.534 | .301 |  | 11.725 | .000 | .181 | .126 |  |  | 2.936 | 4.131 |  |  |  |
|  | Empathy | .018 | .014 | .349 | 1.261 | .210 |  |  | .103 | 9.741 | -.010 | .045 | -.229 | .123 | .112 |
|  | Manipulation | -.457 | .381 | -.191 | -1.199 | .233 |  |  | .311 | 3.219 | -1.212 | .299 | -.087 | -.117 | -.106 |
|  | Gender | -.482 | .383 | -.201 | -1.260 | .210 |  |  | .308 | 3.244 | -1.241 | .277 | -.193 | -.123 | -.112 |
|  | Empathy^Manipulation | -.047 | .017 | -.647 | -2.734 | .007 |  |  | .141 | 7.102 | -.081 | -.013 | -.385 | -.259 | -.243 |
|  | Gender^Manipulation | .219 | .500 | .081 | .439 | .662 |  |  | .230 | 4.354 | -.772 | 1.211 | -.230 | .043 | .039 |
|  | Gender^Empathy | -.013 | .016 | -.194 | -.820 | .414 |  |  | .141 | 7.105 | -.046 | .019 | -.144 | -0.80 | -.073 |
|  | Empathy^Manipulation^Gender | .021 | .022 | .199 | .959 | .340 |  |  | .182 | 5.481 | -.022 | .063 | -.266 | .094 | .085 |

**Table S6:** Regression analysis with creativity in the brick task as the dependent variable, but without the Gender factor

| Model | | Unstandardized Coefficients | | Standardized Coefficients | t | Sig. | R^2^ | Adj. R^2^ | Collinearity Statistics | | 95% CI for B | | Correlations | | |
| --- | --- | --- | --- | --- | --- | --- | --- | --- | --- | --- | --- | --- | --- | --- | --- |
|  |  | B | Std. Error | Beta |  |  |  |  | Tolerance | VIF | LB | UP | Zero-order | Partial | Part |
| 1 | (Constant) | 3.187 | .160 |  | 19.936 | .000 | .060 | .043 |  |  | 2.870 | 3.504 |  |  |  |
|  | Empathy (raw score) | -.011 | .005 | -.229 | -2.467 | .015 |  |  | 1.00 | 1.00 | -.021 | -.002 | -.229 | -.230 | -.229 |
|  | Manipulation (rude/polite) | -.211 | .222 | -.088 | -.950 | .344 |  |  | 1.00 | 1.00 | -.651 | .229 | -.087 | -.091 | -.088 |
| 2 | (Constant) | 3.185 | .152 |  | 20.965 | .000 | .159 | .136 |  |  | 2.884 | 3.486 |  |  |  |
|  | Empathy | .004 | .006 | .071 | .584 | .560 |  |  | .523 | 1.913 | -.009 | .016 | -.229 | .056 | .052 |
|  | Manipulation | -.211 | .211 | -.088 | -1.00 | .319 |  |  | 1.000 | 1.000 | -.630 | .207 | -.087 | -.096 | -.088 |
|  | Empathy^Manipulation | -.032 | .009 | -.435 | -3.563 | .001 |  |  | .523 | 1.913 | -.049 | -.014 | -.385 | -.324 | -.314 |

**Table S7:** Regression analysis with flexibility in the brick task as the dependent variable

| Model | | Unstandardized Coefficients | | Standardized Coefficients | t | Sig. | R^2^ | Adj. R^2^ | Collinearity Statistics | | 95% CI for B | | Correlations | | |
| --- | --- | --- | --- | --- | --- | --- | --- | --- | --- | --- | --- | --- | --- | --- | --- |
|  |  | B | Std. Error | Beta |  |  |  |  | Tolerance | VIF | LB | UP | Zero-order | Partial | Part |
| 1 | (Constant) | 3.447 | .201 |  | 17.134 | .000 | .076 | .050 |  |  | 3.048 | 3.846 |  |  |  |
|  | Empathy (raw score) | .008 | .005 | .168 | 1.605 | .111 |  |  | .778 | 1.286 | -.018 | .002 | -.237 | -.153 | -.148 |
|  | Manipulation (rude/polite) | -.148 | .214 | -.064 | -.692 | .491 |  |  | 1.000 | 1.000 | -.571 | .276 | -.060 | -.066 | -.064 |
|  | Gender (male/female) | -.336 | .242 | -.145 | -1.385 | .169 |  |  | .777 | 1.287 | -.816 | .145 | -.223 | -.132 | -.128 |
| 2 | (Constant) | 3.443 | .362 |  | 13.101 | .000 | .172 | .125 |  |  | 2.922 | 3.964 |  |  |  |
|  | Empathy | .005 | .010 | .099 | .469 | .640 |  |  | .175 | 5.702 | -.016 | .025 | -.237 | .046 | .042 |
|  | Manipulation | -.130 | .327 | -.056 | -.396 | .693 |  |  | .393 | 2.544 | -.778 | .519 | -.060 | -.039 | -.035 |
|  | Gender | -.363 | .360 | -.157 | -1.009 | .315 |  |  | .324 | 3.083 | -1.078 | .351 | -.223 | -.098 | -.090 |
|  | Empathy^Manipulation | -.029 | .010 | -.419 | -2.928 | .004 |  |  | .384 | 2.602 | -.049 | -.009 | -.381 | -.275 | -.260 |
|  | Gender^Manipulation | -.026 | .474 | -.010 | -.054 | .957 |  |  | .238 | 4.207 | -.966 | .915 | -.241 | -.005 | -.005 |
|  | Gender^Empathy | .002 | .010 | .037 | .241 | .810 |  |  | .332 | 3.013 | -.018 | .023 | -.133 | .024 | .021 |
| 3 | (Constant) | 3.595 | .290 |  | 12.383 | .000 | .184 | .129 |  |  | 3.020 | 4.171 |  |  |  |
|  | Empathy | .015 | .013 | .317 | 1.146 | .254 |  |  | .103 | 9.741 | -.011 | .042 | -.237 | .112 | .102 |
|  | Manipulation | -.335 | .367 | -.145 | -.912 | .364 |  |  | .311 | 3.219 | -1.062 | .393 | -.060 | -.089 | -.081 |
|  | Gender | -.464 | .369 | -.201 | -1.258 | .211 |  |  | .308 | 3.244 | -1.195 | .267 | -.223 | -.122 | -.111 |
|  | Empathy^Manipulation | -.045 | .017 | -.649 | -2.749 | .007 |  |  | .141 | 7.102 | -.078 | -.013 | -.381 | -.260 | -.244 |
|  | Gender^Manipulation | .083 | .482 | .032 | .171 | .864 |  |  | .230 | 4.354 | -.872 | 1.037 | -.241 | .017 | .015 |
|  | Gender^Empathy | -.012 | .016 | -.182 | -.769 | .443 |  |  | .141 | 7.105 | -.043 | .019 | -.133 | .075 | -.068 |
|  | Empathy^Manipulation^Gender | .025 | .021 | .253 | 1.221 | .225 |  |  | .182 | 5.481 | -.016 | .067 | -.230 | .119 | .108 |

**Table S8:** Regression analysis with flexibility in the brick task as the dependent variable, but without the Gender factor

| Model | | Unstandardized Coefficients | | Standardized Coefficients | t | Sig. | R^2^ | Adj. R^2^ | Collinearity Statistics | | 95% CI for B | | Correlations | | |
| --- | --- | --- | --- | --- | --- | --- | --- | --- | --- | --- | --- | --- | --- | --- | --- |
|  |  | B | Std. Error | Beta |  |  |  |  | Tolerance | VIF | LB | UP | Zero-order | Partial | Part |
| 1 | (Constant) | 3.267 | .154 |  | 21.173 | .000 | .060 | .042 |  |  | 2.961 | 3.573 |  |  |  |
|  | Empathy (raw score) | -.011 | .004 | -.237 | -2.551 | .012 |  |  | 1.00 | 1.00 | -.020 | -.003 | -.237 | -.237 | -.237 |
|  | Manipulation (rude/polite) | -.142 | .214 | -.061 | -.662 | .509 |  |  | 1.00 | 1.00 | -.567 | .283 | -.060 | -.063 | -.061 |
| 2 | (Constant) | 3.265 | .147 |  | 22.154 | .000 | .150 | .126 |  |  | 2.973 | 3.557 |  |  |  |
|  | Empathy | .002 | .006 | .050 | .408 | .684 |  |  | .523 | 1.913 | -.009 | .014 | -.237 | .039 | .036 |
|  | Manipulation | -.142 | .205 | -.061 | -.0693 | .490 |  |  | 1.000 | 1.000 | -.548 | .264 | -.060 | -.067 | -.061 |
|  | Empathy^Manipulation | -.029 | .009 | -.415 | -3.386 | .001 |  |  | .523 | 1.913 | -.046 | -.012 | -.381 | -.310 | -.300 |
